# Trimodality Therapy for Bladder Preservation in the Elderly Population with Invasive Bladder Cancer

**DOI:** 10.3389/fonc.2014.00206

**Published:** 2014-08-05

**Authors:** Guy-Anne Turgeon, Luis Souhami

**Affiliations:** ^1^Department of Oncology, Division of Radiation Oncology, McGill University Health Centre, Montreal, QC, Canada

**Keywords:** bladder cancer, elderly, trimodality therapy, radiation therapy, chemotherapy, bladder preservation

## Abstract

Bladder cancer is considered as primarily a disease of the elderly, typically aged in their 70s or 80s and often with associated medical comorbidities. Unfortunately, fewer elderly patients receive radical treatment for muscle-invasive bladder cancer (MIBC) that their younger counterparts. Over the last decades, several studies have shown that the use of trimodality therapy consisting of transurethral bladder resection followed by concomitant chemotherapy and radiation therapy results in comparable outcomes to radical cystectomy, considered the gold standard for this disease. In this review, we revised the literature on bladder-preservation treatments using the trimodality approach in the elderly population with MIBC.

## Introduction

The growing segment of the elderly population results in an increase incidence of cancers and of patients requiring continuous medical care. This is especially the case for cancers with long latency period, such as urothelial bladder carcinoma, in which the peak incidence is at a late age (1). Worldwide, cancer of the urinary bladder is the ninth most common cancer accounting for a yearly estimation of 386,300 new cases and 150,200 deaths (2). Most of these cancers are diagnosed when superficial, but 25% of bladder cancers are invasive at diagnostic, a stage at which the lesion has a high lethal potential if left untreated.

In uro-oncology, the underutilization of curative bladder therapies in the elderly population is a consensus. Current data show that 23–35% of patients aged between 70 and 80 years do not receive curative therapy and for those aged over 80 this rate increases to 35–55% (3–6). This aged population is often affected by multiple competing comorbidities, potentially limiting tolerance to a curative surgical treatment. Radical cystectomy, considered the gold standard treatment for muscle-invasive bladder cancer (MIBC), can be compromised due to changes in patients’ physiological reserves and functional status influencing physicians toward less aggressive and less effective treatment approaches. Although most surgical series include a proportion of aged patients, these are usually well selected and healthier subjects and are not representative of the entire elderly bladder cancer population.

Over the last several years, numerous prospective studies (7–16) have shown that trimodality therapy (TMT) – involving maximally feasible transurethral resection of the bladder tumor (TURBT) followed by concomitant chemotherapy (CT) and radiation therapy (RT) – for MIBC results in comparable overall survival (OS) rates to contemporary surgical series (17–21). Even though chemoradiation (CRT) delivered in a curative manner can be well tolerated by even very elderly patients, there is a general underutilization of this treatment modality among older patients.

This paper reviews the literature on the use of TMT for bladder preservation in the elderly population with MIBC.

## Methods

Data for the present review were identified by a structured search of the following databases: MEDLINE (via OvidSP 1946 to March 5, 2014; via PubMed 1946 to March 5, 2014); Embase Classic + Embase (via OvidSP 1947 to March 5, 2014). The search strategy used textwords and relevant indexing to describe the following concepts: “bladder/urothelial cancer,” “chemoradiotherapy,” “bladder preservation/combined modality/trimodality,” and “elderly/aging/aged/octogenarian/geriatric.” Search terms were modified as necessary to accommodate for differences in indexing across databases. Search results were limited by language of publication to English or French. Full papers and conference abstracts were considered, but only publications reporting specifically on elderly cohorts treated in majority with concomitant CRT were included. Publications reporting no survival data (22, 23) and studies including patients younger than 65 years old (24–27) or patients with metastatic disease (24) were excluded.

## Results

Eight publications and four conference abstracts, including a total of 496 elderly patients, met our eligibility criteria. The studies include one prospective comparative phase II trial (28), two case series from prospective trials (29, 30), and nine retrospective analysis (31–39).

### Demographics

The patients’ demographics are presented in Table 1. The 12 studies included from 14 to 93 elderly patients treated with curative intent, bladder-preservation approach between the years 1985 and 2013. An age ≥ 70 years was the most common elderly definition, used in half of the studies. Overall, the median reported age was 78 years and 76% of the population was male. Urothelial carcinoma corresponded to the majority of cases (>95%), with more than eight studies including exclusively patients diagnosed with urothelial carcinoma. Presence of hydronephrosis was reported by half of the authors in which it was diagnosed in 17–40% of the patients, while complete pre-treatment TURBT showed large inter-study variability ranging from 14 to 79% in the latest publications. Overall, 10% of this elderly population had a poor performance status before treatment with an ECOG of 2–3 or a KPS ≤ 60, whereas at least 24% were operative candidates whom decided to undergo bladder-preservation therapy. Tumors were predominantly stage T2–T3. Patients with recurrent superficial lesions (Ta-T1) were occasionally included (5% of cases), and 21 patients (4%) were clinically staged N1–3. In all studies, computed tomography was the standard staging imaging modality.

**Table 1:** **Patient demographics and treatment parameters**.

Authors [year]	Study (tx years)	*n*	Elderly definition	Median age (year) [range]	Clinical stage	Histology	Hydronephrosis	Complete TURBT	Induction treatment	Concurrent chemotherapy	Radiation dose (Gy)
Arias et al. (31) [1997]	Retrospective (1988–1994)	20	≥70 years	74 [70–78]	T2–4,N0–1,M0	Urothelial	40%	35%	TURBT + MVAC	CDDP	65
Eapen et al. (29) [1998]	Prospective data (cases) (1985–1996)	35	≥75 years	– [75–88]	Ta-4,N0–1,M0	–	31%	14%	TURBT (80% pts)	IA CDDP	60
Pfister et al. (32) [2000]	Retrospective (–)	45	Elderly and frail	75 [68–86]	T1–3,N0,M0	–	–	–	TURBT	CDDP	50
Goffin et al. (33) [2004]	Retrospective (1985–2000)	14	≥70 years	79 [74–87]	T2–3,N0,M0	Urothelial Squamous	–	43%	TURBT	CDDP ± 5-FU CDDP ± MCV(adj.)	Group 1: 52–60 Group 2: 24.0 + 20.0 (3 Gy bid) Group 3: 40.8 + 24.0 (1.6–1.8 bid)
Tran et al. (34) [2009]	Retrospective (1992–2005)	39 3 pts RT alone	≥70 years	78 [70–87]	T2–T4a,N0,M0	Urothelial	21%	64%	TURBT	CDDP ± 5-FU CDDP ± MCV(adj.) 5-FU	Group 1: 52–64 Group 2: 24.0 + 20.0 (3 bid) Group 3: 40.8 + 24.0 (1.6–1.8 bid)
Rodica et al. (35) [2009] *Abstract*	Retrospective (2005–2007)	34	Elderly	79 [66–89]	T2–T4, Nx, M0	Urothelial	–	–	–	GEM	mean: 56.4
Khoury et al. (36) [2011] *Abstract*	Retrospective (1996–2007)	68	Elderly and frail	78 [70–91]	T1–T4a,Nx,M0	Urothelial (85%)	–	Dx TURBT only	None	CDDP CBDCA	median: 63
Hsieh et al. (37) [2011]	Retrospective (2006–2009)	19 10 pts RT alone	≥65 years	79 [65–90]	T1–T4,N0–2,M0	Urothelial	–	–	TURBT	CDDP ± 5-FU CBDCA ± GEM	median: 57.6 IMRT or HT
Beltran et al. (38)[2012] *Abstract*	Retrospective (2010–2011)	16 4 pts RT alone	≥78 years	83 [78–88]	T2–T4,N0–1,M0	Urothelial	25%	–	TURBT ± CBDCA + GEM	CBDCA CDDP	median: 65
Azuma et al. (28) [2013]	Prospective (1997–2013)	134 OMC = 89 RC = 45	≥70 years	OMC = 77 [70–91]	Tis-3,N0,M0	Urothelial	–	–	TURBT	IA CDDP + HD	60.4
Clayman et al. (30) [2013] *Abstract*	Prospective data (cases) (1986–2008)	93	≥75 years	–	T2–T4a,N0,M0	Urothelial	– (mostly none)	– (approx 2/3)	TURBT ± MCV	CDDP ± 5-FU CDDP + taxol	40 + 24–25 (qd or bid)
Turgeon et al. (39) [2014]	Retrospective (2008–2012)	24	≥70 years	79 [72–88]	T2–3,N0,M0	Urothelial	17%	79%	TURBT	GEM ± Everolimus CDDP	50 (2.5Gy qd) IMRT

*5-FU, 5-fluorouracil; adj., adjuvant; bid, twice daily; CBDCA, carboplatin; CDDP, cisplatin; GEM, gemcitabine; Gy, gray; HD, hemodialysis; HT, helical tomotherapy; IA, intra-arterial; IMRT, intensity-modulated radiation therapy; MCV: methotrexate, cisplatin and vinblastine; MTX, methotrexate; MVAC, methotrexate, vinblastine, doxorubicin, cisplatin; *n*, number of patients; OMC, Osaka Medical College; pts, patients; qd, once daily; RC, radical cystectomy; RT, radiotherapy; Squamous, squamous cell carcinoma; taxel, paclitaxel; TURBT, transurethral resection of bladder tumor; Dx, diagnostic; tx, treatment; Urothelial, urothelial carcinoma; –, data not available*.

### Treatments

Table 1 summarizes treatment details. Pre-treatment TURBT was a common practice except for one series in which CRT without TURBT was purposely investigated (36). Accounting for all studies, 3% of the patients did not receive concurrent CRT and were treated by TURBT followed by RT alone. Neoadjuvant CT with methotrexate, cisplatin, and vinblastine (MCV) with or without doxorubicin (MCVA) or the combination of carboplatin and gemcitabine was used in approximately 7% of the elderly population. Cisplatin was the most common drug given concomitantly with RT, administered to 77% of the patients, mainly alone but occasionally in combination with 5-fluorouracil (5-FU) or paclitaxel. Gemcitabine or carboplatin were given concomitantly with RT to 58 and 38 patients, respectively. CT schedule administration and doses varied between studies. Similarly, RT regimens also differed but most patients received a minimum of 60 Gy to the bladder. RT was usually delivered to the whole pelvis followed by a boost to the tumor area. Split-course RT was used in 37% of the patients. Conventional two- and three-dimensional RT techniques were used in all studies except in two case series in which the use of intensity-modulated radiation therapy (IMRT) was investigated (37, 39). Two studies used intra-arterial instead of intra-venous concurrent cisplatin administration (28, 29).

### Outcomes

The overall median follow-up was 23 months (Table 2). Based on eight studies, the median complete response (CR) rate was 72% [range: 38–93%]. The two papers proposing intra-arterial CT administration reported the highest CR rates at 91 and 93%.

**Table 2:** **Median follow-up, outcomes, and toxicities**.

Authors [year]	Median follow-up (months)	Complete response %	Cancer-specific survival % (years)	Overall survival % (years)	Acute grade 3–4 toxicities	Late grade 3–4 toxicities
Arias et al. (31) [1997]	60 [30–93]	55	79 (2)	75 (2)	10% (GU)	5% (Cystectomy 2nd to hemorrhagic cystitis)
			54 (3)	34 (3)	
			43 (5)	27 (5)	
Eapen et al. (29) [1998]	16 [3–123]	93	82 (2)	72 (2)	≈29% (pneumonia, stroke, GI, neuropathy)	23% (death, stroke, enteritis, sacralgia)
			63 (3)	43 (3)	
			63 (5)	34 (5)	
Pfister et al. (32) [2000]	–	51	–	50 (2)	–	–
Goffin et al. (33) [2004]	17	–	–	45 (2)	43% (GI, GU, HEM, Heart failure)	0%
Tran et al. (34) [2009]	15 [2–126]	77	60 (2)	44 (2)	28% (GI, GU, HEM)	8% (GU)
			48 (3)	39 (3)	
			37 (5)	30 (5)	
Rodica et al. (35) [2009] *Abstract*	17	38	–	82 (1)	24% (GI, HEM)	–
Khoury et al. (36) [2011] *Abstract*	55	–	–	50 (2)	19% (GU, HEM, Renal)	7% (GU, GI, Recto-vesical fistula)
				31 (5)	
Hsieh et al. (37) [2011]	–	–	–	33 (2)	16% (HEM)	–
Beltran et al. (38) [2012] *Abstract*	14	–	–	73 (1)	0%	–
Azuma et al. (28) [2013]	38 [4–189]	91	–	93 (2)	0%	–
				88 (3)	
				88 (5)	
Clayman et al. (30) [2013] *Abstract*	59 (all MGH cohort)	67	60 (5)	–	–	–
Turgeon et al. (39) [2014]	28 [7–60]	83	80 (2)	69 (2)	17% (GI,GU, HEM, Liver)	0%
			71 (3)	61 (3)	

*GI, gastroinstestinal; GU, genitourinary; HEM, hematologic; MGH, Massachusetts General Hospital; –, data not available*.

Survival data were heterogeneously reported. Cancer-specific survival (CSS) data were available from four papers and one abstract, including a total of 211 patients. The median 2-, 3-, and 5-year CSS rates were 80% [60–82%], 59% [48–71%], and 52% [37–63%], respectively. OS data were more frequently available, with 11 of the 12 studies reporting their results. The median 2-, 3-, and 5-year OS rates were 50% [33–93%], 43% [34–88%], and 31% [27–88%], respectively (Table 2).

### Toxicities

Data from seven studies reported that full-dose CRT was completed in 55% of the patients, and up to 85% of the patients completed CRT but with CT dose reduction. Grade 3–4 acute toxicity ranged from 0 to 43% and grade 3–4 late toxicity ranged from 0 to 23%. The most common acute toxicities were gastrointestinal (GI), genitourinary (GU), and hematologic (Table 2). A single death (pneumonia) and a single treatment-related cystectomy due to hemorrhagic cystitis were reported.

## Discussion

### Undertreatment of the elderly

The definition of “elderly” is variable in the literature. The most popular definition was dogged by our western society governments using the chronological age of 65 years to determine eligibility to retirement pension. The literature variability for the elderly definition is well sampled in urologic studies. In our 12 studies, chronological age ≥ 65, ≥ 70, and ≥ 75 years was used to define elderly. Considering that the decline in functional status is specific to each individual, labeling someone as elderly simply by age and depriving such person of a potential curative therapy must be done with extreme caution, particularly considering that life expectancy has increased over the last several decades. Superimposed to the progressive physiologic and functional deterioration seen with aging (40), many other variables may affect the ability of the patient to tolerate therapy, including competing medical comorbidities, social, and psychological status as well as family dynamics.

The decision-making on curative versus palliative treatment in the elderly population diagnosed with MIBC is challenging. Using the National Cancer Data Base of patients diagnosed with MIBC between 2004 and 2008, Gray et al. (5) reported that only 35% of patients aged 81–90 years were treated with curative intent (cystectomy or definitive RT or CRT), while less than 15% of those aged >90 years received similar approach. Disappointingly, while their proportion of patients treated with radical cystectomy decreased with increasing age, the percentage of patients treated with definitive CRT did not increase proportionally, letting place to observation. Recently, Noon et al. (6) reviewed their experience on cancer-specific mortality (CSM) and competing other-cause mortality in patients diagnosed with bladder cancer and reported that a higher CSM was seen in the elderly population compared to a younger cohort. In their population, only 12% of patients aged >80 years were treated with cystectomy or radical RT. This higher CSM in the elderly was felt to be due to undertreatment and by the prevalence of higher risk tumors in this population at the time of treatment (6, 41). Higher risk tumors would result from late recognition of warning signs and symptoms, decrease response of the host’s immune system, decrease efficacy of intravesical Bacille Calmette–Guerin (42), and the tendency of physicians to defer curative treatment in older individuals until all alternatives have been exhausted. Two recent studies (43, 44) of RC also reported a higher CSM in older individuals even after adjusting for pathological staging.

### Radical cystectomy in the elderly population

Weizer (45) reviewed the surgical outcomes of RC in elderly patients with MIBC. The authors estimate at 40% the proportion of operated elderly individuals with high anesthetic risk, and 40–50% of these patients with cardiovascular or pulmonary comorbidities. In his review, the 30- and the 90-day peri-operative mortality range were 0–9.5% and 0–11%, respectively.

Even in a healthy younger individual, the bladder removal, the lymphadenectomy, the potential blood loss, and the anesthesia are large physiological stresses creating substantial challenges to the organ systems. The complication rate from RC in conventional large series is 24–64%, many of which are related to the complex procedures of the urinary diversion. For the elderly patients, due to decreased physiologic reserves compromising their recoverability, higher complications rates are to be expected. Others (46) have not shown major differences in complication rates when patients aged >80 years are compared to their younger counterparts. Thus, chronological age alone should not necessarily exclude patients from a RC when this procedure is the best therapeutic option, provided that a proper patient selection, based particularly in adequate functional status, is carried out prior to the surgical intervention.

### TMT bladder preservation in the elderly population

Prospective trials of bladder-preservation TMT in MIBC (12, 15, 16) show equivalent outcomes to large RC series (17, 18, 20, 21), with 5-year OS rates in the range of 48–57%. Furthermore, both treatments have shown to be curative alternatives also available to elderly patients diagnosed with MIBC. However, the potential treatment-related complications are to be taken seriously. Surgical series have shown a relatively high incidence of peri-operative morbidity and mortality in elderly patients undergoing RC (45, 47), and TMT trials have shown that acute grade 3 or higher hematologic or GI toxicities are not infrequent (12, 15, 48, 49). Thus, particularly in this frail population, potentially serious acute and late toxicity post-TMT is a valid concern.

The 12 studies included in our review are heterogeneous, but they all individually concur that curative intent TMT bladder preservation in elderly patients is feasible, relatively well tolerated, and, as for younger patients, it seems to lead to outcomes equivalent to RC. Acknowledging the limitations of our review, which includes small, retrospective studies and data reported only in abstract form, the large number of elderly patients treated and the consistent positive outcomes reported strongly suggest that most elderly patients should be considered for curative TMT regardless of their chronological age.

Of the 496 patients studied, all were aged ≥65 years, one quarter was diagnosed with hydronephrosis, and more than 50% had incomplete pre-treatment TURBT. Furthermore, approximately two-thirds were not surgical candidates and, at least, 10% had poor performance status. Patients’ comorbidities were not available in most studies. These patients’ and tumor’s characteristics do not fit the optimal criteria for TMT. However, this population is likely to represent the reality of the elderly population diagnosed with MIBC. These patients are often considered borderline to receive aggressive treatment, but for whom palliation would be sub-optimal management. Since they most often do not fit the TMT eligibility criteria, elderly patients are not well represented in large prospective trials. The difficulty in determining the optimal treatment for this population goes further. Studies involving only elderly patients are highly affected by other-cause mortality resulting in short median follow-up and consequently less conclusive outcomes. This was noticed in our review in which half of the studies reported median follow-up of less than 2 years.

### Improved survival

The perception that the risk–benefit ratio of elderly patients managed with radical treatment was too high has been disclaimed by evidence that elderly patients have significant survival advantage when treated aggressively. In 2004, Hollenbeck et al. (50) demonstrated that RC, RT alone and TURBT alone in patients older than 80 years significantly reduced the risk of death from bladder cancer compared to watchful waiting.

In our reviewed datasets, the median 5-year CSS of elderly patients was 52% [37–63%]. This outcome is certainly comparable to the 5-year CSS of 28–68% reported in the elderly surgical review by Weizer (45). Clayman et al. (30), on a retrospective review from the Massachusetts General Hospital, compared the results of an elderly population treated with TMT for MIBC to their younger counterparts. The 5-year CSS rate of the elderly population was slightly lower than the younger patients but it did not reach significance (60 versus 69%; *p* = 0.2). They also reported that the elderly group had a tendency toward a lower CR rate (67 versus 77%; *p* = 0.07) and received significantly less full dose of consolidation CT then the younger cohort (59 versus 77%; *p* = 0.009).

### Toxicities

Curative intent TMT bladder preservation is certainly feasible and tolerable in the elderly population. The range of 0–43% of acute toxicity represents well the acute toxicity rates found in most TMT prospective treatment regimens (7, 8, 11–14, 49). In terms of late toxicities, the RTOG pooled analysis (48) reported that 7% of the patients experienced late grade 3 pelvic toxicity, with 5.7% GU and 1.9% GI toxicities. They describe that typically these toxicities appear within 2 years of treatment, last a median of 7 months, and rarely persist. The overall reported rate of grade 3–4 late toxicity in our review was 6% [0–23%], which is similar to the literature.

### Decreased toxicity by intensity-modulated radiation therapy

In a previous study by our group (34), we reported that only 33% of patients received full-dose concurrent radiosensitizing CT and their acute grade 3–4 overall and GI toxicities were 23 and 15%, respectively. Following this experience, our group started a multidisciplinary TMT program for elderly patients using hypofractionated IMRT (39). Notwithstanding some differences in treatment parameters, patient selection and also acknowledging that comparison of results across different studies has inherent limitations and cannot be considered definitive, when comparing our last results with our previous experience, we observe a clear improvement in outcomes with the number and grade of acute and late toxicities declining considerably. In the IMRT-treated patients, 79% completed their concurrent CT, acute grade 3–4 hematologic toxicity occurred in 13% of the patients, and acute grade 3 GI or GU toxicity in only 4% of the patients. The use of IMRT in TMT bladder preservation in elderly patients was also reported in 2011 by Hsieh et al. (37) In their small population treated with IMRT, no grade 3–4 acute GI or GU toxicity occurred, but hematologic toxicity was seen in 26% of patients. In both studies, IMRT demonstrated a superior dose sparing of normal organs, without a detrimental effect on local control of the disease, leading to a lower toxicity rate in comparison to other studies using two- or conventional three-dimensional RT.

### Optimal radiosensitizing chemotherapy

Cisplatin is the most active single agent against urothelial bladder carcinoma and has been frequently used as a radiosensitizer in MIBC. Unfortunately, its use is frequently limited due to deteriorating renal function in the elderly.

Alternative regimens of radiosensitizing CT have been tested. Carboplatin has occasionally replaced cisplatin for “unfit” patients. However, significant inferior outcomes have been reported with this agent (12, 36), and it should not be considered an optimal substitution for cisplatin. Newer chemotherapeutic agents, particularly gemcitabine and paclitaxel, appear to be interesting alternatives for the elderly population. Both agents used alone have shown significant activity against urothelial tumors and are potent radiosensitizers. Gemcitabine was used in two prospective TMT trials recruiting surgical candidates. Both trials showed CR rates as high as 88 and 91%, with excellent CSS (51, 52). In another study, Borut and Lijana (53) in a phase I trial investigating non-surgical patients with median age 74 years, obtained a 3-year OS of 64%. In the three trials, GI toxicity was the limiting factor. A recently closed, randomized phase II trial (RTOG 0712) will attempt to confirm these results by comparing gemcitabine with the combination of cisplatin and 5-FU (54).

Paclitaxel was successfully used as radiosensitizer in RTOG 0233 (55) and RTOG 99-06 studies (14). In a poor-risk patient population, paclitaxel was used twice weekly with concomitant RT. All evaluable patients achieved a CR with a 3-year OS of 40% (56). In a phase I–II trial (RTOG 0524), non-surgical elderly patients received paclitaxel (41 patients) with or without transtuzumab (21 patients overexpressing HER2/Neu). Although the CR rate for the combination was encouraging, the associated toxicity may make it unacceptable for this challenging population (57).

The combination of 5-FU and mitomycin C (MMC) in addition to RT has been shown to be of value, as compared to RT alone, in the BC2002 randomized trial (49). A significant improvement in locoregional control was seen favoring the synchronous combination with no significant increase in toxicity. The authors chose 5-FU and MMC based on their previous phase I and II data (58), to allow the inclusion of “less fit” patients. The more permissive eligibility criteria included patients with a creatinine clearance greater than 25 mL/min, with a diagnosis of hydronephrosis and/or with an ECOG performance status of 2. Consequently, the median age of their cohort was 72 years, which is representative of the true bladder cancer population.

### Importance of the quality of life

The bladder cancer literature focuses on CSS and OS, providing almost no information on patients’ quality of life and function, which is often of greatest importance for the elderly community. Patients treated with RC, experience significant changes in urinary and sexual functions, accompanied with psychological and relationship stresses. Integrity and body image has also shown to be significantly affected (59). Furthermore, elderly patients are less likely to undergo neobladder reconstruction (45–47, 59) because of the higher peri-operative morbidity and mortality risks of the complex procedure, and due to the relatively high probability of functional problems requiring long-term increased care and potentially compromising the patient’s independence and social life. Patients older than 75 years old, treated with orthotopic neobladder were reported to have daytime and nighttime continence of only 56 and 25%, respectively, which is worse than younger populations (60).

Despite advances in surgical technique, most patients would prefer to maintain their native bladder if oncologic outcomes were uncompromised. The rationale for organ preservation strategies has been to improve quality of life while achieving comparable long-term survival. In the latest TMT bladder-preservation trials, about three-quarters of surviving patients maintain their native bladder (12, 15, 39, 61). The function of the urinary bladder and of the surrounding organs at risk (the bowel, the rectum, the anal sphincter, the lymph vessels as well as the nerves, and vessels involved in sexual function) may nevertheless be altered during and after irradiation. In 2002, Henningsohn et al. (62) compared long-term survivors of MIBC, aged ≥65 years and treated with either RC or full-dose RT alone. Using anonymous postal questionnaires, the authors showed that individuals treated with RT had better sexual function than patients treated with RC. Furthermore, 74% of the long-term survivors treated with RT were found to have a preserved organ function with little or no urinary distress. Earlier, Caffo et al. (63), in a mixed aged cohort, also reported that quality of life was better in their bladder-preservation group compared to their RC group. The poorer quality of life in the RC patients was accentuated by the stoma, by a lack of sexual activity, and by a worsened physical condition. For the bladder-preservation group, there was a low incidence of urinary symptoms and an acceptable sexual adjustment. The two groups’ social and recreational activities were not significantly affected. Overall, the improved quality of life with bladder preservation in comparison to RC appears to be a valid consideration.

## Conclusion

For an elderly patient, the decision to undergo curative treatment is often a trade-off between a potential loss of function and extension of life. Daily radiation treatments can be inappropriate for older patients while other treatment strategies could result in a shift toward greater physical or psychological dependence. To avoid such endings and be able to evaluate how a patient’s condition may affect his ability to respond to treatment and tolerate potential complications, the treating physician must recognize that elderly patients have unique medical, social, and functional needs compared with younger patients.

This review shows that curative intent bladder-preservation TMT for elderly patients diagnosed with MIBC is feasible and well tolerated. Furthermore, in elderly patients, bladder-preservation TMT seems to lead to outcomes equivalent to radical cystectomy, while minimizing the risks of treatment morbidity, allowing organ preservation and offering an improved quality of life potential. Newer RT technology is now used and can lead to decrease toxicity. The inclusion of new molecular markers may improve patient selection by identifying those most likely to benefit from bladder preservation or RC (64–66). Physicians should not deny patients potentially curative treatment based solely on chronological age.

## Conflict of Interest Statement

The authors declare that the research was conducted in the absence of any commercial or financial relationships that could be construed as a potential conflict of interest.
